# Etiology of Colitis-Complex Diarrhea in Growing Pigs: A Review

**DOI:** 10.3390/ani11072151

**Published:** 2021-07-20

**Authors:** Farhad M. Panah, Charlotte Lauridsen, Ole Højberg, Tina Skau Nielsen

**Affiliations:** Department of Animal Science, Aarhus University, AU Foulum, DK-8830 Tjele, Denmark; farhadm.panah@anis.au.dk (F.M.P.); ole.hojberg@anis.au.dk (O.H.); tinas.nielsen@anis.au.dk (T.S.N.)

**Keywords:** colonic inflammation, adult pigs, biomarkers, dietary strategies, specific colitis, non-specific colitis

## Abstract

**Simple Summary:**

Diarrhea in growing pigs is a challenge for the pig industry since it is associated with reduced animal welfare, retarded growth, increased feed conversion ratio, and is often treated with antibiotics. One of the major causes of diarrhea in the growing period is large intestinal inflammation, often referred to as colitis. The exact causes of colitis-complex diarrhea are still to be understood, but dietary factors and/or pathogens have been recognized as the major factors in developing colitis-complex diarrhea. In this review, a thorough picture of pathogens, dietary factors, and a number of possible biomarkers related to colitis-complex diarrhea is presented.

**Abstract:**

Colitis-complex diarrhea (CCD) in pigs can be defined as a type of diarrhea, which is associated with colonic inflammation and disrupted colonic gut barrier functionality in growing pigs (4–16 weeks post-weaning). It is a challenge for the pig industry as it is associated with the high use of antibiotics, reduced animal welfare, and depressed growth rate. The exact etiology of CCD is still unclear; however, pathogens including *Brachyspira (B.) hyodysenteriae, B. pilosicoli*, and swine whipworms such as *Trichuris (T.) suis* have been involved in specific colitis (SC). In the absence of specific pathogens, dietary factors, such as high levels of protein, pelleted feedstuffs, and lack of sufficient antioxidants, can result in non-specific colitis (NSC). On the other hand, supplement of polyunsaturated fatty acids (PUFA) and polyphenols, sufficient supply of essential amino acids (e.g., threonine, cysteine, and proline), short-chain fatty acids (SCFA; especially butyrate), and resistant starch have shown to confer preventing/ameliorating effects on CCD. Different putative biomarkers associated with CCD have been presented. It is anticipated that a comprehensive picture of the possible causes of CCD and potential dietary interventions could cast light on the direction of future studies aimed at developing preventive and curative strategies against CCD in growing pigs.

## 1. Introduction

Diarrhea in pigs can in general be characterized by increased frequency of defecation of abnormally liquid feces accompanied by the increased quantity of excreta and reduced fecal dry matter (DM) content [1,2]. Diarrheal diseases in pigs are mostly multifactorial and could be due to hypersecretion, malabsorption, hypermobility, and increased permeability of gut epithelium [3,4]. It was previously suggested that the mere presence of pathogens was not necessarily the cause of diarrhea in pigs, demanding the existence of other factors [1] including social and physical stresses, e.g., mixing pigs with non-littermates and reduced room temperature at weaning [4]. Moreover, dietary factors can also play a part in developing diarrhea, which could be evident during the shift from sow’s milk to solid feed and high content of non-starch polysaccharides (NSP) in the diet [1,5]. Moreover, banning of antibiotic growth promoters from pig production leads to more frequent occurrence of diarrhea, also in the growing period [6].

Also termed as ‘grower scour’ [7], colitis-complex diarrhea (CCD) is referred to as a type of diarrhea, which is due to colonic inflammation (colitis) and typically occurs in weeks 4–16 post-weaning (during the growing period). CCD is a challenge for the swine industry as it is associated with the immense use of therapeutic antibiotics (due to larger body weight compared to the weaning period) and the following biosafety and economical costs on pig farms [8,9]. CCD may be caused by feed factors, management [5], and/or a wide variety of pathogens, e.g., *Brachyspira* (*B.*) *hyodysenteriae* [10], *B. pilosicoli* [11], *Salmonella* (*S.*) *enterica* serovar Typhmurium [12], *Escherichia* (*E.*) *coli.* [2], more rarely *Lawsonia* (*L.*) *intracellularis* [13,14], and swine whipworms [15]. Pigs affected with CCD are most likely to have decreased feed conversion efficiency, poorer growth, higher mortality, and increased susceptibility to other diseases with consequent lower market and breeding values [5]. Overall, CCD is typically classified in two categories: (1) specific colitis (SC), caused by one or more specific pathogens, (2) non-specific colitis (NC), mainly caused by dietary factors without the involvement of an identified pathogen.

Defined as colonic inflammatory disease, porcine colitis may share some of the pathogenic mechanisms of human inflammatory bowel disease (IBD), e.g., ulcerative colitis (UC) [5]. Bowel wall thickening with increased vascularization (with maintenance or loss of wall stratification) is considered the most widely used diagnostic criterion for the characterizing of human IBD [16]. Therefore, to understand CCD in pigs, results from human-based studies will also be presented.

Since CCD in growing pigs is a multifactorial disease, having a better understanding of factors involved in its etiology could help promote preventive and curing strategies. Therefore, the objective of this review is to describe pathogens and dietary factors involved in the development of CCD, to present putative biomarkers for identification of CCD, and possible dietary strategies aimed at preventing/attenuating CCD in growing pigs older than six weeks.

## 2. Colonic Epithelium Function

### 2.1. Colonic Ion Exchange

**Figure 1:**
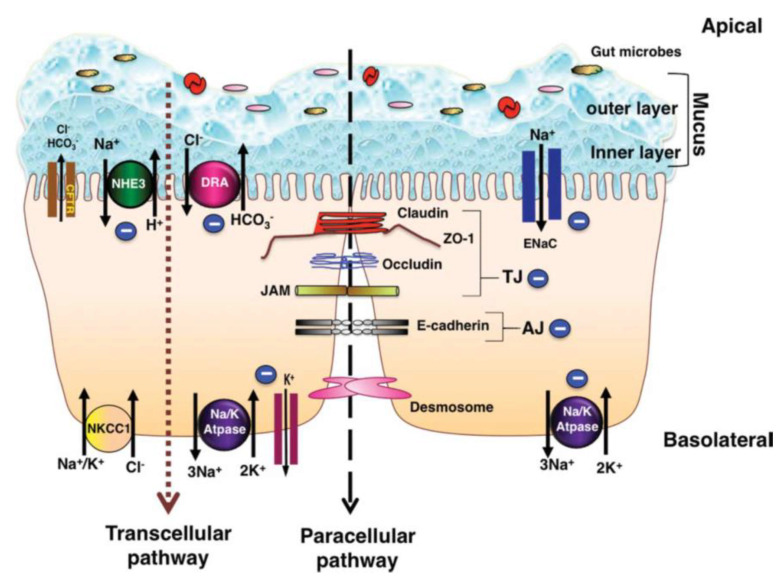
Schematic pathways of colonic ion transportation, adopted from Anbazhagan et al. [20].

The pathogenesis of diarrhea related to inflammation in the colon is a multifactorial event [17]. Understanding the ion-absorption/secretion mechanisms in the colon and the effects of inflammatory mediators on epithelial transport function is therefore of great importance. Figure 1 depicts the major ion transportation pathways in the colon. Generally, normal stool is low in Cl^−^ and Na^+^ and high in K^+^ since reabsorption of Na^+^ and secretion of K^+^ (both active and passive) takes place in the colon [3]. The reason for the low level of Cl^−^ in normal stool is that short-chain fatty acids (SCFA) produced in the colon can replace Cl^−^, while luminal concentration of HCO_3_^−^ is similar to its concentration in plasma [3]. In the colon, Na^+^ gets absorbed through the stimulatory effect of SCFA, through an aldosterone-sensitive sodium absorption by the epithelial Na^+^ channel (ENaC) in the distal colon [18], and through an Na-H exchange parallel to a Cl-HCO_3_^−^ exchange (in the proximal colon) which is responsible for an electro-neutral Na-Cl absorption [19]. SCFA are the primary anions in the lumen, which also contribute to Na absorption through apical Na-H, Cl-SCFA, and SCFA-HCO_3_^−^ exchanges; this type of Na^+^ absorption is not hampered by cyclic adenosine monophosphate (cAMP) [19]. Secondary messengers, such as cAMP, cyclic guanosine monophosphate (cGMP), intracellular calcium (Ca^+^), and neurohumoral substances, can activate Na-H exchange (NHE) genes such as NHE3, which is involved in neutral absorption of sodium [18]. However, increased mucosal cAMP and intracellular Ca^2+^ concentration in the colon can inhibit apical Na-H and Cl-HCO_3_^−^ exchanges and reduce absorption of Na^+^ and Cl^−^, consequently reducing the water absorption [19]. For apical exchange of Cl-HCO_3_^−^ in the colon, downregulation in adenoma (DRA or SLC26A3) is the major exchanger, which is a chloride-sulfate anion transporter in the upper crypt and surface epithelium of the colon [17,20]. DRA activity was shown to be inhibited by increased cellular cAMP and Ca^2+^ [21]. Moreover, the gene expression of DRA was reported to be heavily diminished in colonic inflammation as an effect of the interleukin-1β (IL-1β) cytokine, hence hampering Cl^−^ absorption [17].

In animal studies, it has been shown that some bacterial pathogens, such as *Salmonella typhimurium* [22], *Shigella dysenteriae* toxin type 1 [23], and *Campylobacter jejuni,* increase intracellular concentrations of Ca^2+^, resulting in inhibition of NHE3 and stimulation of an excessive secretion of Na^+^ and Cl^−^ [18]. Therefore, impaired Na^+^ absorption and stimulation of Ca^2+^ secretion can result in diarrhea [18]. Excessive intracellular secretion of Ca^2+^ could be a tertiary effect of microbial pathogenesis since pathogens first stimulate the enteric nervous system, then increase the release of neurotransmitters and, ultimately, enhance the secretion of Ca^2+^ [23]. Increased levels of luminal ions can be expected as a result of inhibition of Na^+^ and Cl^−^ absorption along with stimulation of excessive Cl^−^ secretion after disruption of the colonic mucus layer by either pathogen- and/or feed-induced inflammatory mediators [4]. Quantification of recovered Cl^−^ from fecal samples [24] of affected pigs can potentially be an indicator of an inflamed colonic epithelium of pigs with colitis.

### 2.2. Cytokines and Luminal Function

Cytokines are small peptide molecules acting as important mediators in the regulation of the immune and inflammatory responses produced by epithelial cells, endothelial cells, and fibroblasts [25]. Cytokines such as interferon-γ (IFN-γ) can directly alter the epithelial tight junctions and increase the transepithelial permeability [25]. In an inflamed colon, cytokines, such as IL-1β [17], IFN-γ, and tumor necrosis factor-α (TNF-α) [26], are culprit keys involved in perturbing the absorption of Na^+^ and Cl^−^ and consequently the water absorption [20], giving rise to stool water content. Overexpression of these cytokines in the colonic mucosa can also cause mucosal damage and dysfunction, which may lead to diarrhea [27]. The increased ionic level in the lumen can reciprocally disturb the absorptive/secretive balance and cause diarrhea in two distinct ways: (1) by increasing the further secretion of ions upon the induced imbalanced electrical charge, and (2) by increasing the extracellular osmotic pressure, resulting in more water diffusion from the enterocytes.

Aside from ion transportation, the intestinal epithelium performs a barrier function through enterocytes and their encircling tight junctions (zona occludens), which limits the passive flow of luminal contents into the blood and lymphatics and the other way around [3]. The restriction of tight junctions is higher in the colon vs. small intestine, and this restriction increases from the proximal to the distal colon [3]. In human patients with Crohn’s disease (CD), the epithelium in inflamed intestinal segments is characterized by a reduction of tight junction strands, strand breaks, and alterations of tight junction protein content and composition [28]. In patients with ulcerative colitis (UC), micro-erosions caused by upregulated epithelial apoptosis as well as a remarkable increase in claudin-2 are the main reasons for early epithelial leaks [28]. The mucosal inflammation in UC increases the permeability of colonic epithelium by changing the tight junctions, which could be a contributing reason to diarrhea appearing due to colonic inflammation [19,29].

### 2.3. Mucins

In addition to the epithelial integrity and the presence of commensal bacteria, the non-immune intestinal barrier is due to mucus production [30]. Mucins are gel-forming high-molecular-weight glycoproteins, which are synthesized and secreted by goblet cells and also act as an important feature for gut barrier functionality [31]. In the intestinal tract, MUC2 is the predominant secretory mucin, and it is the main structural component of the colonic mucus layer [32]. Perturbed synthesis and secretion of mucin by the goblet cells, in response to pathophysiological alterations in the intestinal mucosa, result in changes in the mucus gel [33]. In the absence of inflammation, the mucus layer is about 700 μm thick [34], whereas exposure to specific bioactive factors, such as hormones, inflammatory mediators, and microbial factors, such as lipopolysaccharides (LPS), flagellin A, and lipoteichoic acids, can upregulate mucin production, in particular MUC2 [35]. Upregulation of mucins such as MUC5AC and MUC4 along with mucus hypersecretion of goblet cells were also shown to be associated with inflammatory diseases of the epithelium [36].

## 3. Colitis

**Figure 2:**
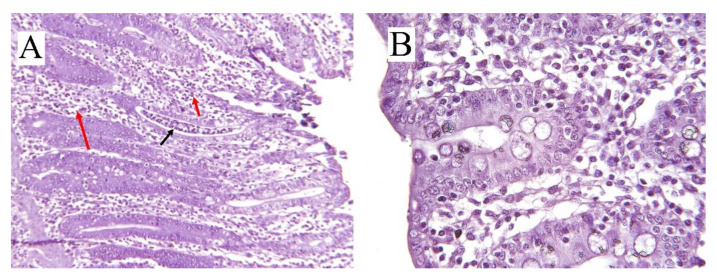
(**A**) Cross-sectional histology (hematoxylin/eosin-stained) of inflamed colon in pig (8 weeks old) with ×10 scale of magnification. Infiltration of neutrophils (black arrow) and mononuclear cells (red arrows) in the crypts can be seen. (**B**) Colonic cross-sectional histology of a healthy pig (11 week) with ×25 scale of magnification.

Inflammation is a constant ongoing process of a normally functioning colon as the healthy colon is continuously offsetting inflammatory responses as a result of exposure to a wide variety of, for example, bacteria in the lumen, dietary antigens, and toxins [27]. If the inflammatory responses go beyond a point to jeopardize the luminal integrity, it can result in diarrhea by increasing gut permeability [37] and impairing its absorptive functionality [4]. Colitis is referred to as inflammation in the colon, which is a consequence of a complex biological defense mechanism against harmful stimuli such as pathogenic bacteria and physical damages [38]. Diarrhea is a non-specific sign of colitis occurring in the acute phase of colitis [39] and for pigs commonly referred to as CCD. This type of diarrhea is typically non-hemorrhagic (in severe cases, hemorrhagic) mucoid diarrhea and infiltration of neutrophils (Figure 2) in the inflamed colon of growing pigs (4–16 weeks post-weaning) [8,40].

However, in younger pigs (e.g., during nursery and post-weaning stage), the main reason for diarrhea is enterotoxigenic *Escerichia* (*E.*) *coli* (ETEC) with fimbriae types F4 and F18 [1,41]. CCD occurs if the epithelia’s barrier function is compromised by loss of epithelial cells or disruption of tight junctions (Figure 1); in that scenario, hydrostatic pressure in blood vessels and lymphatics will cause water and electrolytes, mucus, protein, and in some cases, red and white cells to accumulate luminally [3]. The bottleneck of colitis diagnosis is the examination of postmortem lesions and histological test (hematoxylin and eosin staining) on the colonic epithelium (Figure 2) [41,42]. In the case of acute or chronic inflammation or necrosis of the colonic mucosa, exudative diarrhea occurs, which is characterized by an increase in fluid production, excretion of the inflammatory products such as serum proteins, and a reduction in absorption of fluids and electrolytes [1]. Unlike the small intestine, the colonic absorptive function is related to the epithelial intercrypt surface instead of villi, while the immature epithelial cells of the crypts perform the secretory function as in the small intestine [4].

### 3.1. Specific Colitis

The most important etiology and epidemiology of common forms of SC (i.e., when one or more pathogens are involved) are briefly presented in Table 1, which will be discussed in the following sections.

#### 3.1.1. Swine Dysentery

Swine dysentery (SD) is a severe mucohemorrhagic diarrheal colitis caused by *B. hyodysenteriae,* formerly known as *Treponema hyodysenteriae* and *Serpulina hyodysenteriae*, a Gram-negative and flagellated anaerobic spirochete [10,50]. Together, the abundance of goblet cells (as a matrix and source of nutrients) and the anaerobic environment in the large bowel contribute to a favorable environment for *B. hyodysenteriae*, confided to the colon [50]. Colonization of the large intestine (cecum and colon) by *B. hyodysenteriae* results in lysis of mucosal cells by bacterial virulence factors, such as hemolysins and proteases, degradative enzymes, and inflammation [2]. Bacterial proteases were reported to be involved in malabsorption of carbohydrates by inactivating the oligosaccharidases of the brush border [3], and the damages to the intestinal wall could aid proliferation of other pathogens [13]. The incidence of SD occurs in pigs during 6–18 weeks of age [13], and the first clinical symptom is excretion of watery or semisolid feces, which progresses as diarrheal feces with large amounts of mucus and variable amounts of blood [51]. Physiologically, SD is associated with dehydration, acidosis, hyperkalemia and, in severe cases, death [51]. Nonetheless, the apparent prevalence of *B. hyodysenteriae* in Danish herds was shown to be relatively low: 2.5% [52].

*Brachyspira hyodysenteriae* is associated with producing a cytotoxic hemolysin as a virulence determinant, which, concurrent with other anaerobic colonic bacteria such as *Fusobacterium necrophorum* or *Bacteroides vulgatus*, can synergize the development of SD in growing pigs [13,50]. The colonic goblet cells of pigs with severe clinical signs of SD may show a substantial increase in the levels of MUC2 mucin and de novo expression of MUC5AC mucin and increased *B. hyodysenteriae,* binding ability to the epithelial mucus [53]. This indicates that morphological changes such as increased mucin production in the colon induced by *B. hyodysenteriae* are mechanisms to facilitate the further infection by increasing the binding sites for *B. hyodysenteriae* attachment. Therefore, increased expression of MUC2 in the inflamed colon of pigs and subsequent higher levels in feces may potentially be considered as contributing biomarkers for the diagnosis of colonic inflammations (Table 2). The consequent increased turnover rate of intestinal mucosal cells could be a reason for increased crypt depth [54] observed in pigs with colitis [42]. However, the genes encoding MUC2 were remarkably downregulated in crypt cells of pigs infected with *Lawsonia (L.) intracellularis* [36], an obligate intracellular, Gram-negative, and microaerophilic pathogen, which infects cells of the ileal epithelium (small intestine) [2]. Therefore, characterization of the variations in mucin expression of the pig colon may be considered as a diagnostic tool for inflammatory diseases such as colitis and discriminating ileitis from colitis.

An indicator of infectious diarrhea is the presence of secretory and pro-inflammatory mediators, such as histamine, prostaglandins, and 5HT (5-hydroxytryptamine or serotonin) as well as proteases released from the mast cells [4,55]. However, *B. hyodysenteriae* can be involved in diarrhea by causing mucosal inflammation in the large intestine upon its attachment, malabsorption by impairing Na^+^, and water-absorptive functionality of the colon epithelium [4], resulting in an increased accumulation of water, Na^+^, Cl^−^, HCO_3_^−^, and K^+^ in the luminal fluid [51]. As a consequence of disrupted absorptive functionality, acidosis and increased serum’s K^+^ could potentially be used as a diagnostic factor in SC [51]. In addition, Cl^−^ is a key-secreted ion that facilitates the transmucosal movement of both Na^+^ and water, which is defused across the paracellular space and into the lumen under the effect of electrical and osmotic gradient [4]. Since glucose, galactose, and amino acid are co-transported with Na^+^ into the colonocytes, their luminal accumulation as a result of SD is expected. Consequently, due to an osmotic gradient caused by undigested carbohydrates [4] in the lumen, more water diffusion could be anticipated; hence, exacerbated diarrhea in infected pigs can occur. This mode of action was attributed to *B. hyodysenteriae,* in addition to the fact that it has no pernicious effects on the intestinal secretory processes and solely disrupts the absorptive function of the epithelium [51].

According to Carr et al. [13], a noteworthy difference between the pathogenic etiologies of colitis is that *B. hyodysenteriae* is a strict anaerobe, occurring only in the large bowel (cecum and colon); therefore, the associated lesions of SD are not seen in the small intestine. In this regard, Argenzio [71] showed that the kinetics of glucose-stimulated water absorption were identical between infected and healthy pigs, indicating that SD had no disruptive effect on the small intestine and ileum. Therefore, oral glucose-electrolyte rehydration would be a pragmatic approach in restoring extracellular fluid losses associated with SD [71]. Glucose and amino acids are co-transported with Na^+^, and the existing intracellular Na^+^ gradient is a driving force for amino acids, oligopeptides, and sugar absorption with the following water absorption due to the osmotic pressure, hence rehydration [1,3].

#### 3.1.2. Spirochetal Colitis

Spirochetal colitis or porcine colonic spirochetosis affects pigs aged 4–20 weeks, and the clinical signs commonly appear 10–14 days after mixing and changing the feed to a grower or pelleted diet [11,13,44]. *Brachyspira* *pilosicoli* is the causative pathogen of spirochetal colitis [44] with the same mode of action as *B. hyodysenteriae* on the absorptive function of the colonocytes, albeit histopathologic lesions are less severe with milder non-hemorrhagic diarrhea [4]. *Brachyspira* *pilosicoli* causes inflammation and mucosal damage throughout the cecum and colon (typhlocolitis; [44]), which by reducing the surface area of the large intestine available for absorption causes diarrhea [13]. Gross lesions of spirochetal colitis are associated with diarrhea with loose to watery and in some cases mucoid feces, and mild mucosal reddening and flecks of pus, resulting in diarrhea and reduced growth rate [11,44]. Microscopic signs of this disease could be seen as mild to moderate catarrhal colitis with erosions of the surface epithelium and spirochetal colonization of the colon epithelium [11]. Trott et al. [44] characterized the postmortem signs of *B. pilosicoli* infection as increased size of the colon in infected animals with flaccid and thin wall, filled with watery, slightly mucoid content, and the mucosal surface was covered with small adherent nodules of digesta.

The occurrence of this disease was characterized by the coinfection of the protozoa *Balantidium coli* associated with the mucosa and lamina propria along with *B. pilosicoli* attached by one end to the colonic epithelium, which triggers neutrophilic exocytosis, excess mucus and increased crypt cell mitotic index [44]. A study in 79 Danish pig herds showed an apparent prevalence of *B. pilosicoli* to be 19.0% [52]. In case of SC, the presence of microbially derived LPS in serum could be a reliable biomarker for microbial detections (Table 2) since LPS is a part of the outer membrane of Gram-negative bacteria and induces inflammatory responses [56]. *Brachyspira* LPS contain the lipid A-sugar core of approx. 10–16 kDa, which is the main difference from LPS of other species, and these lipopolysaccharides produced by *B. hyodysenteriae* are speculated to be involved in the colonic damage [57]. In an in vivo study, pigs administered with LPS developed acute phase response (APR) and hepatic production of acute phase proteins, e.g., C-reactive protein (CRP), haptoglobin (HP), and pig major acute phase protein (pig-MAP) as a result of increased stimulation of pro-inflammatory cytokines, such as TNF-α, IL-1β, and IL-6 [58]. Therefore, in case of SC, the seral measurement of LPS, CRP, HP, and pig-MAP could also be considered as putative biomarkers.

#### 3.1.3. Parasitic Colitis

Swine whipworms can cause inflammation in the cecum and spiral colon, resulting in defecation of dark loose stools containing blood and diarrhea [15]. In Danish organic pig farms, with access to pastures, there is a high risk of the exposure of the animal to the parasite eggs, present mainly in the pasture [47]. Among swine parasitic whipworms, *Trichuris (T.) suis* can infect young and growing pigs in both indoor and outdoor production systems [47] that can cause severe mucoid to hemorrhagic diarrhea, dehydration, anorexia, reduced gain, and increased feed conversion ratio [46,72]. The eggs of the parasite are passed in feces from infected animals, and, once eaten, they can travel through the digestive tract, and in the small intestine and cecum, the larva hatches and penetrates the mucosa through the crypts of Lieberkühn in the distal ileum, cecum, and colon [46]. Crypt hyperplasia, goblet cell hyperplasia, and a general hypertrophy of mucosa in the proximal colon in addition to the increased level of parasite-specific antibodies in the serum of pigs infected with *T. suis* in week 10 of age were reported to be associated with *T. suis* infection [45]. Adult worms are highly potent to cascade an inflammatory response in addition to inducing immunosuppressive properties in infected pigs through high release of nitric oxide (NO) and upregulating arginase activity in macrophages [48]. The excessive production of NO in abnormal situations induces inflammation in the colon [73].

Conducting clinical serologic assays for the increased level of 20 kDa excretory/secretory glycoprotein antigen in the serum of infected pigs could be used for diagnosis of *T. suis* [74]. Hygienic practices to prevent the fecal-oral transmission of the eggs, including regular removal of feces and organic debris, could be considered as preventive strategies, and, once infected, pigs could be treated with anthelmintic [72].

### 3.2. Non-Specific Colitis and Role of Dietary Factors in Inducing Colitis

When no specific pathogens can be identified, the term NSC is designated to describe this form of colitis [5,75]. As shown in Table 1, NSC mainly affects growing pigs (12–40 kg, approx. 4–12 weeks post-weaning), and it has been estimated to affect 40–80% of pigs in the UK and other European countries [41,49]. Non-specific colitis is characterized by subacute colitis with mucosal hyperplasia, mononuclear cell infiltration, multifocal mucosal erosions, colonic lesions of increased crypt depth, and poor growth rate [41,42], mainly because of the impaired absorptive functionality and dehydration due to diarrhea. Hyperplasia in colon-associated lymphatic tissue, shining mucosa in colon, and mesenteric lymph node hyperplasia without any related pathogens detected in fecal samples were reported to be considered as the gross pathological signs of NSC [42]. As mentioned earlier, both SC and NSC induce morphological changes in the colon due to the cascaded inflammatory responses. Knowing the intermediate and possible end-products of these inflammatory mechanisms could be used as possible biomarkers for facilitating the diagnosis of colitis in diarrheal pigs. Calprotectin and lactoferrin are proteins derived from activated neutrophils, and since they are quite stable in feces and could be detected by quantitative ELISA, they are considered as inexpensive and non-invasive biomarkers for colitis in humans [61]. Calprotectin and lactoferrin have been reported to be closely correlated to the inflammations in patients with IBD when diagnosed by endoscopic measures [62]. Similarly, in case of colonic inflammation in the pigs, infiltration of neutrophils in the inflamed sites has been reported [42] for which fecal recovery of calprotectin and lactoferrin could be expected as a promising biomarker of colitis. However, only a few studies have been done on adopting this method for identifying colitis in pigs [60,63].

The exact etiology and epidemiology of NSC is generally poorly understood [5,42]. However, several dietary factors seem to be implicated in the development of NSC (Table 3), and these dietary factors may also facilitate pathogenic infections in the large intestine and cause a synergized SC. For instance, NSC was reported to be more prevalent in fast-growing pigs fed high-density diets, i.e., diets with high-metabolizable energy [13] and the presence of trypsin inhibitors in peas, beans, and soya, and deficiency of vitamin E has also been associated with the occurrence of NSC [13]. Feeding diets poor in tryptophan, as a precursor of niacin (vitamin B3) biosynthesis, has also been related to the incidence of NSC [49]. For the biosynthesis of niacin out of tryptophan, the presence of riboflavin, vitamin B_6_, and iron is required to be in adequate amounts. Urinary excretion of N-methylnicotinamide is a reliable indicator for nicotinic acid deficiency [76]. It was reported that sufficient dietary supplementation of niacin (≥15 µg/g feed) could prevent the incidence of NSC and also reverse the condition in pigs [40]. Niacin inhibits inflammation responses by downregulating nuclear transcription factors-κB (NTF-κB, Table 2) signaling pathway in guinea pigs [77] and was shown to reverse colitis in rats challenged by iodoacetamide [78]. Other dietary factors involved in the incidence of NSC could be dietary protein, dietary fiber, and pelleted feedstuff, which will be discussed below.

#### 3.2.1. Dietary Crude Protein and NSC

Histological examination of colonic tissue of pigs with NSC showed the loss of microvilli and apoptosis of surface epithelial cells, which could be due to the increased concentrations of NH_4_^+^, indoles, and phenols arising from increased bacterial fermentation of especially undegraded dietary protein [40,49]. The increased crypt depth related to NSC was also speculated to be a result of protein fermentation in the colon [42]. Moreover, increasing the dietary crude protein content from 17 to 23% (Table 3) in five-week-old pigs was shown to be associated with reduced tight junction genes (e.g., Zonula occludens-1 and Occludin) expression in the gut, resulting in increased gut permeability and diarrhea [37]. In the same study, the incidence of NSC was also related to the increased expression of pro-inflammatory cytokines IL-1β, IFN-γ, and cystic fibrosis transmembrane conductance regulators (CFTR) in the distal colon as a result of increased protein content of the diet [37]. Upregulation of gene expression of inflammatory cytokines such as IL-1β and IL-6 in the proximal colon of pigs can increase the intestinal epithelial permeability by altering tight junction proteins and disrupting the epithelial integrity [90,91]. This reflects the close correlation of these cytokines with the incidence of the colonic inflammation, which could be used as biomarkers for both NSC and SC in pigs. Wu et al. [37] reported an increased concentration of Cl^−^ and CFTR in the digesta of the terminal colon in piglets fed diets with 23% protein vs. 17%, indicating an increased epithelial permeability and excessive secretion of Cl^−^ due to the increased dietary protein. This can be an indication of the disrupted absorptive functionality of the colon epithelium since luminal accumulation of Cl^−^ ions and CFTR is tightly related to the incidence of diarrhea [92], and their quantification in the fecal samples could be used as possible biomarkers for protein-related NSC. An increased level of plasma urea nitrogen in the pigs with diarrhea induced by the increased level of dietary protein was also reported previously [37], which could be another indirect indicator of excessive protein fermentation in the colon. In addition, biogenic amines, NH_4_^+^, indoles, and phenols are byproducts of protein fermentation in the large intestine, and with the recovery assays from the fecal samples, they may also be considered as potential biomarkers of colitis induced by protein fermentation.

#### 3.2.2. Dietary Fiber and Non-Starch Polysaccharides

Even though colonic fiber fermentation is generally considered positive for the host animal (see further in Section 4.3), the extent of dietary fiber (DF) fermentation in the large intestine has an impact on the development of NSC, e.g., fermentation of cellulose and insoluble arabinoxylans in the colon by bacteria was reported to be involved in the incidence of NSC [49]. The effect of DF on the development of NSC depends, to a great extent, on their physicochemical properties such as degree of lignification and their solubility [83]. This is possibly because of the fact that insoluble fibers decrease the mean retention time in the large intestine by stimulating the propulsive colonic motility [93], reducing the fermentation and absorption [83] and thereby possible reduction in total diversity of the microbiota. Soluble fiber in the diet was also shown to be associated with higher colonic fermentation and more microbial colonization [94]. Although its exact underlying mechanism is not understood, it was speculated that soluble fiber can increase viscosity of digesta in the large intestine, providing substrate and attachment matrix for non-commensal microbes [83]. Moreover, hindgut fermentation of DF was speculated to increase the concentrations of potentially toxic components, such as ammonia, indoles, and phenols, arising from increased bacterial DF fermentation [49], in particular fermentation of fiber-associated protein.

Results from studies on Scottish pig farms indicate a strong relationship between the occurrence of NSC and diets high in non-starch polysaccharides (NSP), especially containing (g/kg feed) arabinose (22.1), xylose (34.9), and glucose (44.1) when compared to farms without NSC with 16.6, 26.2, 31.4 g/kg feed, respectively [5]. In a study by Trot et al. [44], it was construed that diets high in NSP and oligosaccharides resulted in increased bacterial fermentation in the large intestine, providing an optimal environment for *B. hyodysenteriae* and *B. pilosicoli* to proliferate in four-week-old pigs. They also observed a mild subclinical colitis in control pigs (not infected) owing to the increased bacterial fermentation in the large intestine. This is a clear indication of the dietary effects as risk factors for the development of SC by inducing NSC beforehand. Feeding diets high in NSP, depending on the fiber source, may reduce the diversity of coliforms in ileo-cecal ostium, mainly due to reduced substrate available for microbial growth; however, this may result in a decreased resistance to “invading pathogens” as a consequence of reduced diversity of the gut total microbiome [95]. Nevertheless, an adaptation after long-term exposure to NSP was reported in the same study, indicating the re-establishment of the pre-existing microbial community in the large intestine. Dietary application of specific enzymes, e.g., *β*-xylanase (4000 μg/kg diet) and the combination of *β*-gluconase (2550 μg/kg diet) and *β*-xylanase (6120 μg/kg diet), was shown to have beneficial effects in controlling the occurrence of NSC in six-week-old pigs [49]. Exogenous enzymes may increase the digestion of the DF fraction of feedstuff, which could otherwise undergo the microbial fermentation in the large intestine. Thomson [49] reported that replacing wheat with barley low in NSP had a positive effect on reducing the incidence of NSC. By doing so, *β*-glucans becomes the predominant DF in the grain fraction instead of arabinoxylans, which appear to exhibit a preventive effect on NSC. Wheat-induced NSC in pigs between 12 and 40 kg is probably related to the presence of the so-called ‘Rye gene’ (1B1R translocation) in specifically genetically modified wheat varieties (Slejpner, Gladiator, Napier, Glasgow, Savannah, Tanker, and Welford), which are of low nutritional value for pigs [49].

#### 3.2.3. Pelleted Feedstuff

Pelleted feedstuff is another dietary factor associated with the increased incidence of NSC, and heat treatment during the pelleting process has been suggested as a causative factor in the development of colitis [5,89]. Pigs are monogastric animals, and they depend on a repertoire of endogenous and, to some extent, exogenous enzymes for feed digestion [96]. The applied heat during the pelleting process may completely or partially inactivate enzymes present in the feed ingredients [97]. This can cause a reduced digestibility of some nutrients, e.g., protein, in the small intestine, which will later be used as substrate for the bacterial fermentation in the large intestine. This could be an explanation of pelleted diets as risk factors for inducing NSC, as fermentation of protein can result in the formation of toxic substances, which can further trigger inflammatory responses in the colon. Pelleted feedstuffs can also result in thriving pathogens such as ETEC F4 during 4–7 weeks of age and result in post-weaning diarrhea [98]. This can infer that dietary factors play a crucial role in the development of the CCD and earlier in the incidence of post-weaning diarrhea by supplying the substrates required for the propagation of pathogens.

## 4. Role of Dietary Factors in Preventing CCD

### 4.1. Polyunsaturated Fatty Acids and Polyphenols

Table 3 presents a summary of the most important dietary factors involved in developing as well as attenuating inflammation in the colon of pigs. Administration of polyunsaturated fatty acids (PUFA), polyphenols, terpeniods, flavonoids, and alkaloids was shown to act as dietary interventions to reduce signaling proteins, e.g., Mitogen-Activated Protein Kinases (MAPKs) involved in increasing pro-inflammatory substances and attenuating nuclear receptors such as nuclear factor kappa light chain enhancer of activated B cells (NF-κB) in pigs [67,99]. Pistol et al. [67] reported that a diet containing 8% grape seed (high in PUFA and polyphenols) ameliorated the colonic inflammation in pigs (three weeks of age) challenged by dextran sulfate sodium (DSS) by inhibiting MAPKs, reducing the nuclear factor kappa B (NF-κB) gene and protein expression, and diminishing the pro-inflammatory cytokines and chemokines production compared to the control group. Among PUFAs, long chain fatty acids ω-3 and ω-6 were suggested to attenuate the IBD progression via reducing the neutrophil transmigration across the intestinal vasculature and the epithelium, preventing the release of pro-inflammatory cytokines and through upregulation of adhesion molecules [66], which can explain their regulatory effect on colonic inflammation. The protective role of PUFA against colonic inflammation in animal models was also reported to be related to their inhibitory effects on the overexpression of pro-inflammatory cytokines [27]. Supplementation of conjugated linoleic acid (ω-6) in the diet of pigs prior to inducing colitis (by *B. hyodysenteriae*) was shown to reduce mucosal damage and maintained the cytokine profile of IFN-γ and IL-10 to the same level as in control pigs [100], indicating a convincing preventive effect of ω-6 on the incidence of colitis, e.g., SD.

Anti-inflammatory effects of polyphenols from grape seed and grape marc extract in pigs were attributed to their modulatory ability on NF-κB and nuclear factor-erythroid 2-related factor-2 (Nrf2), which is a redox-sensitive transcription factor [59] and regulates a wide array of antioxidant responsive elements to protect the body against oxidative stress [101,102]. Unchecked accumulation of reactive oxygen species (ROS) and deficiency in antioxidants can cause an imbalance between pro- and antioxidants (oxidative stress) [38], which was shown to result in damages to the intestinal tissue of pigs, leading to bacterial translocation and compromising the intestinal barrier functionality [103]. Activation of NF-κB both in macrophages and in epithelial cells of inflamed colon [65] reflects the importance of NF-κB in the initiation of inflammation as a response to increased IL-1β and TNF-α [64], and increased ROS and bacterial LPS [59]. In addition to digestive enzymes in the phagocytes, ROS produced by phagocyte cells play a crucial role in destroying the internalized pathogens [104]. In both guinea pig, with trinitrobenzene sulfonic acid (TNBS)-induced colitis, and mice (with DSS-induced colitis), oxidative stress disrupted the mitochondrial function and reduced purine synthesis as well as purinergic neuromuscular transmission, which led to a remarkable decrease in inhibitory junction potential (IJP) in inflamed spots [105]. Inhibitory junction potential is a biphasic inhibitory junction potential of many regions of the gut, which is evoked by inhibitory nerve stimulation [106]. Since factors such as TNF-α, adhesion of pathogens, and activated phagocytes can result in ROS overproduction and oxidative distress [107], evaluation of oxidative stress in growing pigs with diarrhea could also be a way to support identification of inflammatory reactions in the colon. There are several measures to assess the presence of oxidative stress, i.e., both concentrations of antioxidants and oxidative status [38,102]. Total antioxidant capacity (TAC) assays, e.g., ferric-reducing ability of plasma (FRAP) [69], serum level of ROS, and thiobarbituric acid reactive substances (TBARS) [68], are used as biomarkers of oxidative stress. Recently, other indicators of oxidative stress have been suggested, which could be tested on saliva samples such as trolox equivalent antioxidant capacity (TEAC), cupric reducing antioxidant capacity (CUPRAC), FRAP, and advanced oxidation protein products (AOPP) and hydrogen peroxide (H_2_O_2_) for oxidant concentrations [70]. Roberts et al. [105] showed that the free radical scavenger Tempol decreased oxidative stress and protected the IJP from the adverse effects of the consequent inflammation. Enzymes, such as superoxide dismutases, glutathione peroxidases, and catalase, and water- and lipid-soluble antioxidants including glutathione, ascorbate (vitamin C), α-tocopherol (vitamin E), ubiquinol, and β-carotene [79] and polyphenols [80] are among particular antioxidant systems in the body to cope with ROS loads. Therefore, it could be inferred that the dietary implication of antioxidants such as phytic acid [108] and phenolic compounds (e.g., tocopherols and phenolic acids) through activation of Nrf2 and scavenging ROS [109] could help alleviating inflammatory responses due to oxidative distress in pigs with colitis.

### 4.2. Dietary Protein and Essential Amino Acids

As stated previously, fermentation of undigested dietary protein in the colon is considered a risk for development of NSC and CCD in pigs [40,49]. Reducing dietary crude protein from 23% to 19% was reported to be associated with maintaining enteric health in pigs aged three weeks by lowering toxic microbial metabolites such as NH_4_^+^ [82]. Interestingly, pigs fed diets containing 21.0%, 19.5%, 18.0%, 16.5%, and 15.0% crude protein showed no differences in performance if they had received sufficient amounts of essential amino acids supplemented in the synthetic form [81], indicating that if the demand for essential amino acids by the pig is covered, no benefits of additional protein is obtained. For instance, the essential amino acid threonine is involved in maintaining the gut barrier function because of its role as a precursor for synthesizing the intestinal (glyco) proteins such as mucin MUC2 [31]. As discussed earlier, inflammation in the colon is associated with increased mucin production, hence an increased demand for precursors of MUC2 biosynthesis is expected. Hence, more knowledge on the specific mechanisms by which certain amino acids can influence epithelial barrier function and inflammatory reactions is required in relation to CCD. In a rat model of DSS-induced colitis, feeding increased amounts of essential amino acids, such as threonine, cysteine, and proline, as the precursors of mucin increased the synthesis of mucin during inflammation [30]. In human and animal models of IBD, an increased requirement for threonine, serine, and cysteine was reported as the consequence of increased MUC2 production in the colon [30]. Moreover, studying mice with and without genes for expressing MUC2, challenged with DSS, showed that MUC2-deficient mice were more susceptible to DSS compared to mice with MUC2-encoding genes [32].

Taken together, the research in rodents indicates that mucins perform a direct antimicrobial function, and with the ability to opsonize microbes they aid clearance of the intestinal tract [36]. Increasing the level of threonine up to 56 mg/g of total amino acid (18 mg above the requirement) was shown to remarkably increase de novo synthesis of mucin in pigs (six weeks old), hence the luminal threonine concentration acutely influences the mucin synthesis [110]. Therefore, dietary supplementation of threonine and other mucin precursors could also be considered as nutritional remedies to offset the overproduction of mucin and buffer the microbial pathogenesis in inflamed colon.

### 4.3. Dietary Fiber

Regardless of previously mentioned facts on DF and colitis, the beneficial role of SCFA on the colon epithelium should not be overlooked. For instance, feeding diets containing high levels of DF (NSP + lignin; 268 g/kg feed DM) to growing pigs resulted in increased colon length compared to the low DF diet group (59 g/kg feed DM) [111]. Fermentation of DF in the large intestine was shown to improve gut maturation, supplying SCFA for the colon mucosa and hindering the adhesion of pathogenic bacteria to the gut mucosa [83]. In the anaerobic condition of colon, DF undergoes fermentation by the microbes from which SCFA are formed [84]. SCFA can confer bactericidal effects by decreasing gut pH [112]. However, Siba et al. [113] argued that increasing colonic digesta pH and decreasing total SCFA as a result of less carbohydrate fermentation in the large intestine by feeding a highly digestible diet to pigs would reduce chances of colonization by *B. hyodysenteriae* and lower the incidence of SD colitis. They showed that pigs fed highly digestible cooked/cooled rice and animal protein did not show colitis or diarrhea, even though they were challenged with *B. hyodysenteriae,* when compared to pigs receiving cooked rice and lupin, wheat and lupin, or wheat and animal protein. This was confirmed by Pluske et al. [114], who showed that regardless of a lower colonic digesta pH and increased SCFA levels in pigs fed wheat, barley, and Australian sweet lupins, they showed higher incidences of SD when compared to pigs fed diets containing rice, sorghum, and maize as the sole cereal sources. They attributed these results to the relatively higher contents of NSP and resistant starch (RS) in wheat, barley, and Australian sweet lupins as they have higher chances to bypass the small intestinal digestion and undergo microbial fermentation in the colon. Resistant starch is the starch portion with a high amylose:amylopectin ratio [115] with low small intestinal digestibility, thereby being available for fermentation in the large intestine [85]. Prohaszka and Lukács [112] demonstrated that the intensity of the antibacterial effect of SCFA in the colon was not related to the absolute quantity of SCFA but rather to the proportion of non-dissociated SCFA molecules. This proportion increases with low pH (pH < 6.0) and decreases with higher pH values (pH 7.0); so does antibacterial activity of SCFA.

Short-chain fatty acids have stimulatory effects on the blood flow, fluid, and electrolyte uptake in the colon [116], play an important role in maintaining intestinal homeostasis [117], and constitute a considerable part of available energy (11–25% of body energy requirement) for pigs [84,118]. Moreover, SCFA can also be metabolized by colonocytes as a source of energy besides glucose and glutamine from vascular origin [119]. Among SCFA, butyrate confers beneficial effects through a variety of colonic mucosal functions, such as inhibition of inflammation and carcinogenesis, reinforcing various components of the colonic defense barrier, and decreasing oxidative stress [120]. Butyrate is of special importance since it is the most preferred substrate for colonocytes [121] and helps maintain their normal phenotype, and it might have selective antimicrobial effects [83]. Butyrate utilization by colonocytes is an oxidative reaction, which consumes O_2_ to yield high amounts of ketone bodies (acetoacetate and *β*-hydroxybutyrate) and CO_2_ in colonic epithelial cells [119]. By promoting the secretion of digestive enzymes as well as gene expression of digestive enzymes, butyrate was reported to be a facilitator of colonic digestion and nutrient absorption [122]. The presence of butyrate in the colon keeps colonocytes in a metabolic state, characterized by high O_2_ consumption (known as C2-skewed metabolism), which results in epithelial hypoxia (<1% O_2_) and less oxygen diffusion to the colon lumen (maintaining anaerobic condition) [123]. Previous studies showed that the G Protein-Coupled Receptor 109A (GPR109A) can be activated by butyrate [124], niacin [125], and 3-hydroxybutyric acid [126] and mediate anti-inflammatory effects [127]. Binding butyrate to G protein-coupled receptors can maintain the regulatory T-cell pool in the mucosa and hamper the inflammatory responses [123]. As mentioned earlier, SD and spirochetal colitis are associated with hampering disruptive functionality of the colon, which results in increased luminal Na^+^ and Cl- secretion. In this regard, SCFA can also boost the absorptive function of the lumen by increasing the reabsorption of Cl^−^ [3] and triggering Na^+^ absorption through apical Na-H and Cl-SCFA exchanges [19].

Disruption of the gut microbiota could cause a depletion of microbe-derived fermentation products, e.g., butyrate, which promotes a metabolic reorientation of terminally differentiated colonocytes towards a so-called C1-skewed metabolism, resulting in increased lactate release, low oxygen consumption and elevated synthesis of iNOS, an enzyme generating nitric oxide [123], acting as an inflammatory factor [109]. Elevated lactate concentration concurrent with reduced butyrate concentration in fecal samples of patients with UC was suspected to be associated with the occurrence of diarrhea [24]. In addition, it was previously shown that NH_4_^+^, which is produced by the colonic microbiota through deamination of amino acids and hydrolysis of urea, impeded the utilization of butyrate by colonocytes followed by a reduction in the production of CO_2_ and ketone bodies [119]. A high rate of CO_2_ production from butyrate is one of the means for examining the functional activity of the colonic mucosa, both clinically and in vivo [121]. Therefore, it may be expected that increasing colonic butyrate production by fiber fermentation and reduced supply of protein for the colonic microbiota is a robust strategy for maintaining the colonic health of pigs and reducing inflammation and consequent diarrhea. Cooked butylated high-amylose maize starch (HAMSB) was shown to successfully deliver the esterified butyrate to the human colon and enhance the populations of *Parabacteroides distasonis,* and in return *P. distasonis* facilitated releasing butyrate from HAMSB in the colon [128].

An in vitro study confirmed the antibacterial effects of non-dissociated SCFA related to the low pH in pigs’ colon, and the bactericidal activity was observed only at pH 5.6–6.6, whereas at pH above 6.6, this effect waned due to diminished quantity of non-dissociated SCFA [129]. Pluske et al. [114] concluded that microbial fermentation of cabohydrates, e.g., NSP and RS, in the colon overruled the bacteriocidal effects related to increased SCFA quantity and reduced pH level. For instance, feeding growing pigs with a diet high in NSP (240 vs. 77 g/kg of feed DM), based on barley and wheat as the basal cereals and supplemented with beet pulp, was shown to increase the luminal content of SCFA [129]. The low NSP diet (77 g/kg feed DM) showed a tendency to increase the butyrate proportion of total SCFA while the high NSP diet (240 g/kg feed DM) resulted in the highest proportion of acetate [130]. Moreover, increased concentrations of non-dissociated SCFA, especially non-dissociated butyrate and acetate, were shown to lower the total SCFA production owing to the harmful effects of non-dissociated SCFA on SCFA-producing microorganisms [131], indicating a non-selective bactericidal effect of SCFA. Diets high in NSP (with 240 g/kg of feed DM) were reported to be associated with a greater proportion of acetic acid while low NSP diets (77 g/kg of feed DM) tended to increase the butyric acid proportion [130]. The composition of NSP in low-NSP diets was mainly comprised of arabinose, xylose, galactose, glucose, and uronic acid with 14.2, 16.9, 11.6, 24.0, and 7.80 g/kg of feed DM, respectively. However, the high-NSP diet contained 59.7, 32.0, 22.8, 84.4, and 33.1 g/kg of feed DM arabinose, xylose, galactose, glucose, and uronic acid, respectively. Therefore, it can be inferred that fermentation of dietary fiber, depending on the composition of NSP, in the colon could possibly be favoring *B. hyodysentriae* and the development of colitis in two ways: (1) by increasing the content of non-dissociated SCFA, which is also harmful for commensal microorganisms and (2) by supplying the substrates required for thriving the potential pathogens and increasing luminal DM content, which could act as a matrix for attachment and survival of the pathogen [114].

### 4.4. Resistant Starch

Although a few studies have reported the provocative effect of RS on the development of colitis in growing pigs [112,113], a recent review argued that the beneficial effects of RS on gut health outweighs its potential drawbacks [85]. It is a highly fermentable fiber source [86], and feeding retrograded tapioca starch (with up to 50% resistance to small intestinal digestion) to pigs increased the colonic SCFA level, the numbers of the healthy gut-associated butyrate-producing *Faecalibacterium prausnitzii,* and suppressed pathogenic bacteria such as *E. coli* [87]. Overall, RS seems to be able to regulate gut microbiota, structure the gut lumen, gut biochemistry, cell signaling, and circulating inflammatory mediators [88].

## 5. Conclusions

Colitis in growing pigs is a multifactorial disease, characterized by inflammation in the colon, infiltration of neutrophils in the inflamed site, disturbed epithelial integrity, increased gut permeability and often associated with diarrhea, and has given rise to the so-called CCD. Although the exact etiology of CCD remains to be understood, *B. hyodysenteriae*, *B. pilosicoli*, *T. suis*, and/or dietary factors have been recognized as the major reasons for its development. Therefore, CCD is categorized as SC (due to a specific pathogen) and NSC (mainly due to dietary factors). Dietary strategies could possibly help prevent and attenuate CCD, among which reducing dietary protein, insoluble fiber, and pelleted feedstuffs, and increasing the level of natural antioxidants in the diet along with maintaining the sufficient levels of essential amino acids, could be briefly named. Several possible indicators associated with CCD have been proposed; nonetheless, there is a void of studies on the specific biomarkers for CCD, which probably requires both microbial analysis of present pathogenic species in fecal samples in combination with immune and inflammatory parameters and microbial metabolites. Hence, more future research devoted to investigate the pig microbiome and its interaction with the host and nutrition is considered important for the development of dietary strategies for the prevention of CCD and thereby reduction in the use of antibiotics for treating diarrhea in both post-weaned and growing pigs.

## Figures and Tables

**Table 1:** Etiology and epidemiology of CCD in growing pigs according to the causative factors and mechanisms of action.

Type of Colitis	Causative Factor	Affected Site	Pigs Age, Week	Mechanism of Action	Clinical Signs	Gross Lesion	References
SC							
Swine dysentery	*B. hyodysenteriae*	Cecum and colon	6–18	Absorptive dysfunctionality, hemolysins, and degradative enzymes	Loose stool, mucoid, hemorrhagic diarrhea, dehydration, and retarded growth rate	Inflamed epithelium, mucosal damage, hyperplasia of the crypts, and spirochetal attachment	[2,10,13,43]
Spirochetal colitis	*B. pilosicoli*	Cecum and colon	4–20	Absorptive dysfunctionality	Mild non-hemorrhagic, mucoid diarrhea, retarded growth rate	Inflamed epithelium, moderate catarrhal colitis, flaccid and thin luminal wall, appearance of small adherent nodules of digesta	[4,11,13,43,44]
Parasitic colitis	*T. suis*	Cecum and spiral colon	4–10	Stimulation of the epithelium and cascading inflammatory responses by hatched eggs and adult worms	Dark loose stool, mucoid to hemorrhagic diarrhea, dehydration, anorexia, and increased feed conversion ratio	Crypt hyperplasia, goblet cell hyperplasia, a general hypertrophy of mucosa, and presence of bipolar eggs	[15,45,46,47,48]
NSC	Dietary factors	Cecum and colon	4–12	Absorptive dysfunctionality and increased epithelial permeability	Loose and mucoid, non-hemorrhagic diarrhea	Mucosal hyperplasia, mononuclear cell infiltration, multifocal mucosal erosions, increased crypt depth	[13,37,42,49]

**Table 2:** Summary of non-invasive putative biomarkers associated with colonic inflammation.

Biomarkers	Type	Direction	Recovery Site	Causative Factor	Affected Site	Reference
MUC2 and MUC5AC	Mucin	Increased expression	Feces	Colitis, swine dysentry	Large intestine	[31,35,36]
LPS ^1^	Saccharide	Increased expression	Serum	Gram-negative pathogens, e.g., *B. hyodysenteriae*	Small and large intestine	[56,57,58,59]
Calprotectin and lactoferrin	Protein	Increased expression	Feces and serum	Colitis and inflammatory factors	Large intestine	[60,61,62,63]
Na^+^, Cl^−^, HCO_3-_, and K^+^	Ion	Reduced absorption and increased luminal accumulation	Feces	*B. hyodysenteriae*	Large intestine	[4,20,26,51]
TNF-α ^2^, IFN-γ ^3^, IL-1β ^4^, IL-6 ^5^, and IL-10 ^6^	Cytokines	Increased expression	Serum and mucus	Pathogens	Small and large intestine	[17,20,26,37,58,64]
NF-κB ^7^	Protein	Increased expression in macrophages and in epithelial cells	Serum	IL-1β and TNF-α, LPS, and ROS ^8^	Epithelial cells of inflamed colon	[59,64,65,66,67]
CRP ^8^, HP ^9^, and pig-MAP ^10^	Protein	Increased concentration	Serum	LPS, IL-1β, and TNF-α	Epithelial cells of colon, and hepatic cells	[58]
FRAP ^11^, TBARS ^12^, and ROS ^13^	TAC ^14^ assay	Increased expression	Serum	Oxidative stress	Epithelial cells of colon	[68,69]
TEAC ^15^, CUPRAC ^16^, AOPP ^17^, and H_2_O_2_	TAC assay	Increased expression	Saliva	Oxidative stress	Epithelial cells of colon	[69,70]

^1^ Lipopolysaccharides; ^2^ Tumor necrosis factor-α; ^3^ Interferon-γ; ^4^ Interleukin-1β; ^5^ interleukin-6; ^6^ interleukin-10; ^7^ Nuclear factor kappa B; ^8^ C-reactive protein; ^9^ Haptoglobin; ^10^ Pig major acute phase protein; ^11^ Ferric reducing ability of plasma; ^12^ Thiobarbituric acid reactive substances; ^13^ Reactive oxygen species; ^14^ Total antioxidant capacity; ^15^ Trolox equivalent antioxidant capacity; ^16^ Cupric reducing antioxidant capacity; ^17^ Advanced oxidation protein products.

**Table 3:** Dietary factors involved in CCD.

Factor	Level	Effect	Reference
Trypsin inhibitor	High	Increased undegraded protein in large intestine and inflammation and causing NSC ^1^	[13]
Vitamin C and E, glutathione, ubiquinol, polyphenols, and β-carotene	Insufficient	Oxidative distress	[13,67,79,80]
Essential amino acids	Insufficient	Oxidative stress by reducing antioxidant enzymes, reduced mucin production	[30,31,49,81]
Dietary protein	≥23%	Increased undegraded protein in large intestine and inflammation due to NH^4+^, reducing gut barrier function and causing NSC	[37,41,49,82]
Soluble NSP ^2^ and RS ^3^	Increased	Ameliorating/preventive effect on large intestinal inflammation, increased SCFA ^4^, and reduced luminal pH	[83,84,85,86,87,88]
Pelleted diet	-	Reduced endogenous enzymes in feedstuffs and causing NSC	[5,89]

^1^ Non-specific colitis; ^2^ Non-starch polysaccharides; ^3^ Resistant starch; ^4^ Short-chain fatty acids.

## Data Availability

Data sharing not applicable.

## References

[1] Constable P., Hinchcliff K., Done S., Grünberg W., Constable P.D., Hinchcliff K.W., Done S.H., Grünberg W. (2017). 7—Diseases of the Alimentary Tract: Nonruminant. Veterinary Medicine.

[2] Zachary J.F., Zachary J.F. (2017). Chapter 4—Mechanisms of Microbial Infections^1^. Pathologic Basis of Veterinary Disease.

[3] Field M. (2003). Intestinal ion transport and the pathophysiology of diarrhea. J. Clin. Investig..

[4] Moeser A.J., Blikslager A.T. (2007). Mechanisms of porcine diarrheal disease. J. Am. Vet. Med. Assoc..

[5] Chase-Topping M.E., Gunn G., Strachan W.D., Edwards S.A., Smith W.J., Hillman K., Stefopoulou S.N., Thomson J.R. (2007). Epidemiology of porcine non-specific colitis on Scottish farms. Vet. J..

[6] Grave K., Jensen V.F., Odensvik K., Wierup M., Bangen M. (2006). Usage of veterinary therapeutic antimicrobials in Denmark, Norway and Sweden following termination of antimicrobial growth promoter use. Prev. Vet. Med..

[7] Jacobson M., Hård af Segerstad C., Gunnarsson A., Fellström C., Klingenberg K., Wallgren P., Jensen-Waern M. (2003). Diarrhoea in the growing pig—A comparison of clinical, morphological and microbial findings between animals from good and poor performance herds. Res. Vet. Sci..

[8] Pedersen K.S., Johansen M., Angen O., Jorsal S.E., Nielsen J.P., Jensen T.K., Guedes R., Ståhl M., Bækbo P. (2014). Herd diagnosis of low pathogen diarrhoea in growing pigs—A pilot study. Ir. Vet. J..

[9] Rhouma M., Fairbrother J.M., Beaudry F., Letellier A. (2017). Post weaning diarrhea in pigs: Risk factors and non-colistin-based control strategies. Acta Vet. Scand..

[10] Hampson D.J., Burrough E.R., Zimmerman J.J., Karriker L.A., Ramirez A., Schwartz K.J., Stevenson G.W., Zhang J. (2019). Swine Dysentery and Brachyspiral Colitis. Diseases of Swine.

[11] Jensen T., Boye M., Møller K. (2004). Extensive intestinal spirochaetosis in pigs challenged with Brachyspira pilosicoli. J. Med. Microbiol..

[12] Wills R.W. (2000). Diarrhea in Growing-Finishing Swine. Vet. Clin. N. Am. Food Anim. Pract..

[13] Carr J., Chen S.-P., Connor J.F., Kirkwood R., Segalés J. (2018). Pig Health.

[14] Karuppannan A.K., Opriessnig T. (2018). Lawsonia intracellularis: Revisiting the Disease Ecology and Control of This Fastidious Pathogen in Pigs. Front. Vet. Sci..

[15] Lawhorn D.B. (2007). Diarrheal disease in show swine. Tex. FARMER Collect..

[16] Strobel D., Goertz R.S., Bernatik T. (2011). Diagnostics in inflammatory bowel disease: Ultrasound. World J. Gastroenterol..

[17] Yang H., Jiang W., Furth E.E., Wen X., Katz J.P., Sellon R.K., Silberg D.G., Antalis T.M., Schweinfest C.W., Wu G.D. (1998). Intestinal inflammation reduces expression of DRA, a transporter responsible for congenital chloride diarrhea. Am. J. Physiol. Gastrointest. Liver Physiol..

[18] Surawicz C.M. (2010). Mechanisms of diarrhea. Curr. Gastroenterol. Rep..

[19] Binder H.J. (2009). Mechanisms of diarrhea in inflammatory bowel diseases. Ann. N. Y. Acad. Sci..

[20] Anbazhagan A.N., Priyamvada S., Alrefai W.A., Dudeja P.K. (2018). Pathophysiology of IBD associated diarrhea. Tissue Barriers.

[21] Musch M.W., Arvans D.L., Wu G.D., Chang E.B. (2009). Functional coupling of the downregulated in adenoma Cl-/base exchanger DRA and the apical Na+/H+ exchangers NHE2 and NHE3. Am. J. Physiol. Gastrointest. Liver Physiol..

[22] Khurana S., Ganguly N.K., Khullar M., Panigrahi D., Walia B.N.S. (1991). Studies on the mechanism of Salmonella typhimurium enterotoxin-induced diarrhoea. Biochim. Biophys. Acta Mol. Basis Dis..

[23] Kaur T., Singh S., Gorowara S., Ganguly N.K. (1995). Role of enteric nervous system in Shigella dysenteriae type 1 toxin-induced fluid secretion in rabbit ileum. J. Diarrhoeal Dis. Res..

[24] Vernia P., Gnaedinger A., Hauck W., Breuer R.I. (1988). Organic anions and the diarrhea of inflammatory bowel disease. Dig. Dis. Sci..

[25] Madara J.L., Stafford J. (1989). Interferon-gamma directly affects barrier function of cultured intestinal epithelial monolayers. J. Clin. Investig..

[26] Amasheh S., Barmeyer C., Koch C.S., Tavalali S., Mankertz J., Epple H.-J., Gehring M.M., Florian P., Kroesen A.-J., Zeitz M. (2004). Cytokine-dependent transcriptional down-regulation of epithelial sodium channel in ulcerative colitis. Gastroenterology.

[27] Liu Y. (2015). Fatty acids, inflammation and intestinal health in pigs. J. Anim. Sci. Biotechnol..

[28] Schulzke J.D., Ploeger S., Amasheh M., Fromm A., Zeissig S., Troeger H., Richter J., Bojarski C., Schumann M., Fromm M. (2009). Epithelial tight junctions in intestinal inflammation. Ann. N. Y. Acad. Sci..

[29] Caruso R., Lo B.C., Núñez G. (2020). Host–microbiota interactions in inflammatory bowel disease. Nat. Rev. Immunol..

[30] Faure M., Mettraux C., Moennoz D., Godin J.-P., Vuichoud J., Rochat F., Breuillé D., Obled C., Corthésy-Theulaz I. (2006). Specific amino acids increase mucin synthesis and microbiota in dextran sulfate sodium–treated rats. J. Nutr..

[31] Puiman P.J., Jensen M., Stoll B., Renes I.B., de Bruijn A.C.J.M., Dorst K., Schierbeek H., Schmidt M., Boehm G., Burrin D.G. (2011). Intestinal Threonine Utilization for Protein and Mucin Synthesis Is Decreased in Formula-Fed Preterm Pigs. J. Nutr..

[32] Van der Sluis M., De Koning B.A., De Bruijn A.C., Velcich A., Meijerink J.P., Van Goudoever J.B., Büller H.A., Dekker J., Van Seuningen I., Renes I.B. (2006). Muc2-deficient mice spontaneously develop colitis, indicating that MUC2 is critical for colonic protection. Gastroenteritis.

[33] Specian R.D., Oliver M.G. (1991). Functional biology of intestinal goblet cells. Am. J. Physiol. Cell Physiol..

[34] Fuss I.J., Strober W., Mestecky J., Strober W., Russell M.W., Kelsall B.L., Cheroutre H., Lambrecht B.N. (2015). Chapter 81—Ulcerative Colitis. Mucosal Immunology.

[35] Martin C.R., Walker W.A., Gleason C.A., Devaskar S.U. (2012). Chapter 70—Innate and Mucosal Immunity in the Developing Gastrointestinal Tract: Relationship to Early and Later Disease. Avery’s Diseases of the Newborn.

[36] Bengtsson R.J., MacIntyre N., Guthrie J., Wilson A.D., Finlayson H., Matika O., Pong-Wong R., Smith S.H., Archibald A.L., Ait-Ali T. (2015). Lawsonia intracellularis infection of intestinal crypt cells is associated with specific depletion of secreted MUC2 in goblet cells. Vet. Immunol. Immunopathol..

[37] Wu Y., Jiang Z., Zheng C., Wang L., Zhu C., Yang X., Wen X., Ma X. (2015). Effects of protein sources and levels in antibiotic-free diets on diarrhea, intestinal morphology, and expression of tight junctions in weaned piglets. Anim. Nutr..

[38] Lauridsen C. (2019). From oxidative stress to inflammation: Redox balance and immune system. Poult. Sci..

[39] Stege H., Jensen T.K., Møller K., Baekbo P., Jorsal S. (2001). Risk factors for intestinal pathogens in Danish finishing pig herds. Prev. Vet. Med..

[40] Thomson J., Smith W.J., Fowler V.R., Edwards S., Hazzledine M. Non-specific colitis in pigs: Defining the condition. Proceedings of the 17th International Pig Veterinary Society Congress.

[41] Luise D., Lauridsen C., Bosi P., Trevisi P. (2019). Methodology and application of Escherichia coli F4 and F18 encoding infection models in post-weaning pigs. J. Anim. Sci. Biotechnol..

[42] Burrough E.R. (2016). Swine Dysentery: Etiopathogenesis and Diagnosis of a Reemerging Disease. Vet. Pathol..

[43] Stanton T.B. (2006). The Genus Brachyspira. Prokar.

[44] Trott D.J., Huxtable C.R., Hampson D.J. (1996). Experimental infection of newly weaned pigs with human and porcine strains of Serpulina pilosicoli. Infect. Immun..

[45] Kringel H., Iburg T., Dawson H., Aasted B., Roepstorff A. (2006). A time course study of immunological responses in Trichuris suis infected pigs demonstrates induction of a local type 2 response associated with worm burden. Int. J. Parasitol..

[46] Pittman J.S., Shepherd G., Thacker B.J., Myers G.H. (2010). Trichuris suis in finishing pigs: Case report and review. J. Swine Health Prod..

[47] Roepstorff A., Mejer H., Nejsum P., Thamsborg S.M. (2011). Helminth parasites in pigs: New challenges in pig production and current research highlights. Vet. Parasitol..

[48] Leroux L.-P., Nasr M., Valanparambil R., Tam M., Rosa B.A., Siciliani E., Hill D.E., Zarlenga D.S., Jaramillo M., Weinstock J.V. (2018). Analysis of the Trichuris suis excretory/secretory proteins as a function of life cycle stage and their immunomodulatory properties. Sci. Rep..

[49] Thomson J. (2009). Feed-associated colitis of growing pigs and its interaction with enteric infections. Acta Sci. Vet..

[50] Gelberg H.B., Zachary J.F. (2017). Chapter 7—Alimentary System and the Peritoneum, Omentum, Mesentery, and Peritoneal Cavity^1^. Pathologic Basis of Veterinary Disease.

[51] Argenzio R.A., Whipp S.C., Glock R.D. (1980). Pathophysiology of Swine Dysentery: Colonic Transport and Permeability Studies. J. Infect. Dis..

[52] Stege H., Jensen T.K., Møller K., Bækbo P., Jorsal S.E. (2000). Prevalence of intestinal pathogens in Danish finishing pig herds. Prev. Vet. Med..

[53] Quintana-Hayashi M.P., Mahu M., De Pauw N., Boyen F., Pasmans F., Martel A., Premaratne P., Fernandez H.R., Teymournejad O., Maele L.V. (2015). The levels of Brachyspira hyodysenteriae binding to porcine colonic mucins differ between individuals, and binding is increased to mucins from infected pigs with de novo MUC5AC synthesis. Infect. Immun..

[54] Jin L., Reynolds L.P., Redmer D.A., Caton J.S., Crenshaw J.D. (1994). Effects of dietary fiber on intestinal growth, cell proliferation, and morphology in growing pigs2. J. Anim. Sci..

[55] Pothoulakis C., Castagliuolo I., LaMont J.T. (1998). Nerves and intestinal mast cells modulate responses to enterotoxins. Physics.

[56] Pedersen K.S., Kristensen C.S., Nielsen J.P. (2012). Demonstration of non-specific colitis and increased crypt depth in colon of weaned pigs with diarrhea. Vet. Q..

[57] Casas V., Vadillo S., San Juan C., Carrascal M., Abian J. (2016). The Exposed Proteomes of *Brachyspira hyodysenteriae* and *B. pilosicoli*. Front. Microbiol..

[58] Wyns H., Plessers E., De Backer P., Meyer E., Croubels S. (2015). In vivo porcine lipopolysaccharide inflammation models to study immunomodulation of drugs. Vet. Immunol. Immunopathol..

[59] Gessner D.K., Fiesel A., Most E., Dinges J., Wen G., Ringseis R., Eder K. (2013). Supplementation of a grape seed and grape marc meal extract decreases activities of the oxidative stress-responsive transcription factors NF-κB and Nrf2 in the duodenal mucosa of pigs. Acta Vet. Scand..

[60] Lallès J.-P., Fagerhol M.K. (2005). Faecal calprotectin: A non invasive marker of inflammation in pigs. ISAH.

[61] Lamb C.A., Mansfield J.C. (2011). Measurement of faecal calprotectin and lactoferrin in inflammatory bowel disease. Frontline Gastroenterol..

[62] Yamamoto T., Shiraki M., Bamba T., Umegae S., Matsumoto K. (2013). Faecal calprotectin and lactoferrin as markers for monitoring disease activity and predicting clinical recurrence in patients with Crohn’s disease after ileocolonic resection: A prospective pilot study. United Eur. Gastroenterol. J..

[63] Bogere P., Choi Y.J., Heo J. (2019). Optimization of Fecal Calprotectin Assay for Pig Samples. J. Agric. Life Sci..

[64] Jobin C., Hellerbrand C., Licato L.L., Brenner D.A., Sartor R.B. (1998). Mediation by NF-kappa B of cytokine induced expression of intercellular adhesion molecule 1 (ICAM-1) in an intestinal epithelial cell line, a process blocked by proteasome inhibitors. Gut.

[65] Rogler G., Brand K., Vogl D., Page S., Hofmeister R., Andus T., Knuechel R., Baeuerle P.A., Schölmerich J., Gross V. (1998). Nuclear factor κB is activated in macrophages and epithelial cells of inflamed intestinal mucosa. Gastroenterology.

[66] Ungaro F., Rubbino F., Danese S., D’Alessio S. (2017). Actors and Factors in the Resolution of Intestinal Inflammation: Lipid Mediators as a New Approach to Therapy in Inflammatory Bowel Diseases. Front. Immunol..

[67] Pistol G.C., Marin D.E., Rotar M.C., Ropota M., Taranu I. (2020). Bioactive compounds from dietary whole grape seed meal improved colonic inflammation via inhibition of MAPKs and NF-κB signaling in pigs with DSS induced colitis. J. Funct. Foods.

[68] Tan C., Wei H., Sun H., Ao J., Long G., Jiang S., Peng J. (2015). Effects of dietary supplementation of oregano essential oil to sows on oxidative stress status, lactation feed intake of sows, and piglet performance. BioMed Res. Int..

[69] Schuh S., Muller L.K., Campos L.P., Moresco R.N., Baldissera M.D., de Oliveira S.C., Campigotto G., Da Silva A.S., Paiano D. (2016). Effect of supplementation of newborn piglets with spray dry blood plasma on weight gain and serum biochemical variables. Comp. Clin. Path..

[70] Rubio C.P., Mainau E., Cerón J.J., Contreras-Aguilar M.D., Martínez-Subiela S., Navarro E., Tecles F., Manteca X., Escribano D. (2019). Biomarkers of oxidative stress in saliva in pigs: Analytical validation and changes in lactation. BMC Vet. Res..

[71] Argenzio R.A. (1980). Glucose-stimulated fluid absorption in the pig small intestine during the early stage of swine dysentery. Am. J. Vet. Res..

[72] Laber K.E., Whary M.T., Bingel S.A., Goodrich J.A., Smith A.C., Swindle M.M., Fox J.G., Anderson L.C., Loew F.M., Quimby F.W. (2002). Chapter 15—Biology and Diseases of Swine. Laboratory Animal Medicine.

[73] Sharma J.N., Al-Omran A., Parvathy S.S. (2007). Role of nitric oxide in inflammatory diseases. Inflammopharmacology.

[74] Hill D.E., Romanowski R.D., Urban J.F. (1997). A Trichuris specific diagnostic antigen from culture fluids of *Trichuris suis* adult worms. Vet. Parasitol..

[75] Taylor-Pickard J.A., Stevenson Z., Glebocka K. (2008). Formula for the Future: Nutrition or Pathology? Elevating Performance and Health in Pigs and Poultry.

[76] Luecke R., McMillen W., Thorp F., Tull C. (1947). The relationship of nicotinic acid, tryptophane and protein in the nutrition of the pig. J. Nutr..

[77] Si Y., Zhang Y., Zhao J., Guo S., Zhai L., Yao S., Sang H., Yang N., Song G., Gu J. (2014). Niacin Inhibits Vascular Inflammation via Downregulating Nuclear Transcription Factor-B Signaling Pathway. Mediat. Inflamm..

[78] Salem H., Wadie W. (2017). Effect of Niacin on Inflammation and Angiogenesis in a Murine Model of Ulcerative Colitis. Sci. Rep..

[79] Thérond P., Bonnefont-Rousselot D., Davit-Spraul A., Conti M., Legrand A. (2000). Biomarkers of oxidative stress: An analytical approach. Curr. Opin. Clin. Nutr. Metab. Care.

[80] Lowe F. (2014). Biomarkers of Oxidative Stress. Syst. Biol. Free Radic. Antioxid..

[81] Toledo J.B., Furlan A.C., Pozza P.C., Piano L.M., Carvalho P.L.O., Peñuela-Sierra L.M., Huepa L.M.D. (2014). Effect of the reduction of the crude protein content of diets supplemented with essential amino acids on the performance of piglets weighing 6–15kg. Livest. Sci..

[82] Nyachoti C.M., Omogbenigun F.O., Rademacher M., Blank G. (2006). Performance responses and indicators of gastrointestinal health in early-weaned pigs fed low-protein amino acid-supplemented diets. J. Anim. Sci..

[83] Molist F., van Oostrum M., Pérez J.F., Mateos G.G., Nyachoti C.M., van der Aar P.J. (2014). Relevance of functional properties of dietary fibre in diets for weanling pigs. Anim. Feed Sci. Technol..

[84] Jha R., Fouhse J.M., Tiwari U.P., Li L., Willing B.P. (2019). Dietary Fiber and Intestinal Health of Monogastric Animals. Front. Vet. Sci..

[85] Regassa A., Nyachoti C.M. (2018). Application of resistant starch in swine and poultry diets with particular reference to gut health and function. Anim. Nutr..

[86] Souza da Silva C., van den Borne J.J.G.C., Gerrits W.J.J., Kemp B., Bolhuis J.E. (2012). Effects of dietary fibers with different physicochemical properties on feeding motivation in adult female pigs. Physiol. Behav..

[87] Haenen D., Zhang J., Souza da Silva C., Bosch G., van der Meer I.M., van Arkel J., van den Borne J.J.G.C., Pérez Gutiérrez O., Smidt H., Kemp B. (2013). A Diet High in Resistant Starch Modulates Microbiota Composition, SCFA Concentrations, and Gene Expression in Pig Intestine. J. Nutr..

[88] Yang X., Darko K.O., Huang Y., He C., Yang H., He S., Li J., Li J., Hocher B., Yin Y. (2017). Resistant Starch Regulates Gut Microbiota: Structure, Biochemistry and Cell Signalling. Cell. Physiol. Biochem..

[89] Thomson J.R., Smith W.J., Murray B.P. (1998). Investigations into field cases of porcine colitis with particular reference to infection with Serpulina pilosicoli. Vet. Rec..

[90] Al-Sadi R., Boivin M., Ma T. (2009). Mechanism of cytokine modulation of epithelial tight junction barrier. Front. Biosci. J. Virt. Lib..

[91] Pearce S.C., Mani V., Boddicker R.L., Johnson J.S., Weber T.E., Ross J.W., Rhoads R.P., Baumgard L.H., Gabler N.K. (2013). Heat stress reduces intestinal barrier integrity and favors intestinal glucose transport in growing pigs. PLoS ONE.

[92] Berger A.L., Ikuma M., Welsh M.J. (2005). Normal gating of CFTR requires ATP binding to both nucleotide-binding domains and hydrolysis at the second nucleotide-binding domain. Proc. Natl. Acad. Sci. USA..

[93] Wilfart A., Montagne L., Simmins H., Noblet J., Milgen J.v. (2007). Digesta transit in different segments of the gastrointestinal tract of pigs as affected by insoluble fibre supplied by wheat bran. Brit. J. Nutr..

[94] Canibe N., Bach Knudsen K.E. (2002). Degradation and physicochemical changes of barley and pea fibre along the gastrointestinal tract of pigs. J. Sci. Food Agric..

[95] Högberg A., Lindberg J.E., Leser T., Wallgren P. (2004). Influence of cereal non-starch polysaccharides on ileo-caecal and rectal microbial populations in growing pigs. Acta Vet. Scand..

[96] Kiarie E.G., Mills A. (2019). Role of Feed Processing on Gut Health and Function in Pigs and Poultry: Conundrum of Optimal Particle Size and Hydrothermal Regimens. Front. Vet. Sci..

[97] Inborr J., Bedford M.R. (1994). Stability of feed enzymes to steam pelleting during feed processing. Anim. Feed Sci. Technol..

[98] Amezcua R., Friendship R., Dewey C., Gyles C. (2002). A case-control study investigating risk factors associated with postweaning Escherichia coli diarrhea in southern Ontario. J. Swine Health Prod..

[99] Somani S.J., Modi K.P., Majumdar A.S., Sadarani B.N. (2015). Phytochemicals and Their Potential Usefulness in Inflammatory Bowel Disease. Phytother. Res..

[100] Hontecillas R., Wannemeulher M.J., Zimmerman D.R., Hutto D.L., Wilson J.H., Ahn D.U., Bassaganya-Riera J. (2002). Nutritional Regulation of Porcine Bacterial-Induced Colitis by Conjugated Linoleic Acid. J. Nutr..

[101] Chen X.L., Dodd G., Thomas S., Zhang X., Wasserman M.A., Rovin B.H., Kunsch C. (2006). Activation of Nrf2/ARE pathway protects endothelial cells from oxidant injury and inhibits inflammatory gene expression. Am. J. Physiol. Heart. Circ. Physiol..

[102] Bacou E., Walk C., Rider S., Litta G., Perez-Calvo E. (2021). Dietary Oxidative Distress: A Review of Nutritional Challenges as Models for Poultry, Swine and Fish. Antioxidants.

[103] Hernandez L.A., Grisham M.B., Twohig B., Arfors K.E., Harlan J.M., Granger D.N. (1987). Role of neutrophils in ischemia-reperfusion-induced microvascular injury. Am. J. Physiol. Heart. Circ. Physiol..

[104] Stuart L.M., Ezekowitz R.A.B. (2005). Phagocytosis: Elegant Complexity. Immunity.

[105] Roberts J.A., Durnin L., Sharkey K.A., Mutafova-Yambolieva V.N., Mawe G.M. (2013). Oxidative stress disrupts purinergic neuromuscular transmission in the inflamed colon. J. Physiol..

[106] Hirst G.D.S., Bywater R.A.R., Teramoto N., Edwards F.R. (2004). An analysis of inhibitory junction potentials in the guinea-pig proximal colon. J. Physiol..

[107] Nathan C., Cunningham-Bussel A. (2013). Beyond oxidative stress: An immunologist’s guide to reactive oxygen species. Nat. Rev. Immunol..

[108] Da Silva E.O., Gerez J.R., Hohmann M.S.N., Verri W.A., Bracarense A.P.F.R.L. (2019). Phytic Acid Decreases Oxidative Stress and Intestinal Lesions Induced by Fumonisin B1 and Deoxynivalenol in Intestinal Explants of Pigs. Toxins.

[109] Rahman I., Biswas S.K., Kirkham P.A. (2006). Regulation of inflammation and redox signaling by dietary polyphenols. Biochem. Pharmacol..

[110] Nichols N.L., Bertolo R.F. (2008). Luminal Threonine Concentration Acutely Affects Intestinal Mucosal Protein and Mucin Synthesis in Piglets. J. Nutr..

[111] Jørgensen H., Zhao X.-Q., Eggum B.O. (1996). The influence of dietary fibre and environmental temoperature on the development of the gastrointestinal tract, digestibility, degree of fermentation in the hind-gut and energy metabolism in pigs. Br. J. Nutr..

[112] Prohaszka L., Lukács K. (1984). Influence of the diet on the antibacterial effect of volatile fatty acids and on the development of swine dysentery. Zent. für Veterinärmed. Reihe B.

[113] Siba P.M., Pethick D.W., Hampson D.J. (1996). Pigs experimentally infected with Serpulina hyodysenteriae can be protected from developing swine dysentery by feeding them a highly digestible diet. Epidemiol. Infect..

[114] Pluske J.R., Pethick D.W., Hopwood D.E., Hampson D.J. (2002). Nutritional influences on some major enteric bacterial diseases of pig. Nutr. Res. Rev..

[115] Åkerberg A., Liljeberg H., Björck I. (1998). Effects of Amylose/Amylopectin Ratio and Baking Conditions on Resistant Starch Formation and Glycaemic Indices. J. Cereal Sci..

[116] Topping D.L., Clifton P.M. (2001). Short-chain fatty acids and human colonic function: Roles of resistant starch and nonstarch polysaccharides. Physiol. Rev..

[117] Parada Venegas D., De la Fuente M.K., Landskron G., González M.J., Quera R., Dijkstra G., Harmsen H.J.M., Faber K.N., Hermoso M.A. (2019). Short Chain Fatty Acids (SCFAs)-Mediated Gut Epithelial and Immune Regulation and Its Relevance for Inflammatory Bowel Diseases. Front. Immunol..

[118] Bergman E.N. (1990). Energy contributions of volatile fatty acids from the gastrointestinal tract in various species. Physiol. Rev..

[119] Darcy-Vrillon B., Cherbuy C., Morel M.-T., Durand M., Duée P.-H. (1996). Short chain fatty acid and glucose metabolism in isolated pig colonocytes: Modulation by NH_4_^+^. Mol. Cell. Biochem..

[120] Hamer H.M., Jonkers D., Venema K., Vanhoutvin S., Troost F.J., Brummer R.-J. (2008). Review article: The role of butyrate on colonic function. Aliment. Pharmacol. Ther..

[121] Roediger W.E.W. (1982). Utilization of Nutrients by Isolated Epithelial Cells of the Rat Colon. Gastroenterology.

[122] Mangian H.F., Tappenden K.A. (2009). Butyrate Increases GLUT2 mRNA Abundance by Initiating Transcription in Caco2-BBe Cells. J. Parenter. Enteral. Nutr..

[123] Litvak Y., Byndloss M.X., Bäumler A.J. (2018). Colonocyte metabolism shapes the gut microbiota. Science.

[124] Thangaraju M., Cresci G.A., Liu K., Ananth S., Gnanaprakasam J.P., Browning D.D., Mellinger J.D., Smith S.B., Digby G.J., Lambert N.A. (2009). GPR109A is a G-protein–coupled receptor for the bacterial fermentation product butyrate and functions as a tumor suppressor in colon. Cancer Res..

[125] Wise A., Foord S.M., Fraser N.J., Barnes A.A., Elshourbagy N., Eilert M., Ignar D.M., Murdock P.R., Steplewski K., Green A. (2003). Molecular identification of high and low affinity receptors for nicotinic acid. J. Biol. Chem..

[126] Taggart A.K., Kero J., Gan X., Cai T.-Q., Cheng K., Ippolito M., Ren N., Kaplan R., Wu K., Wu T.-J. (2005). (D)-β-hydroxybutyrate inhibits adipocyte lipolysis via the nicotinic acid receptor PUMA-G. J. Biol. Chem..

[127] Chen G., Ran X., Li B., Li Y., He D., Huang B., Fu S., Liu J., Wang W. (2018). Sodium Butyrate Inhibits Inflammation and Maintains Epithelium Barrier Integrity in a TNBS-induced Inflammatory Bowel Disease Mice Model. EBioMedicine.

[128] Clarke J.M., Topping D.L., Christophersen C.T., Bird A.R., Lange K., Saunders I., Cobiac L. (2011). Butyrate esterified to starch is released in the human gastrointestinal tract. Am. J. Clin. Nutr..

[129] Prohászka L. (1986). Antibacterial Mechanism of Volatile Fatty Acids in the Intestinal Tract of Pigs agains Escherichia coli. J. Vet. Med. B.

[130] Anguita M., Canibe N., Pérez J.F., Jensen B.B. (2006). Influence of the amount of dietary fiber on the available energy from hindgut fermentation in growing pigs: Use of cannulated pigs and in vitro fermentation. J. Anim. Sci..

[131] Xiao K., Zhou Y., Guo C., Maspolim Y., Ng W.J. (2016). Impact of undissociated volatile fatty acids on acidogenesis in a two-phase anaerobic system. J. Environ. Sci..

